# Supplementary material for: Development of ENTV reverse genetics system and phenotypic evaluation of rescued virus reveals host-specific replication patterns in mosquitoes
Source: bioRxiv. 2025 Jul 29:2025.07.29.667424. Preprint. [Version 1] doi: 10.1101/2025.07.29.667424 (PMC12324323; doi:10.1101/2025.07.29.667424)
Supplement: Supplement 1 — Supplementary Figure 1. The entire 3’ UTR of ENTV was identified. 3’ stem-loop structure of ENTV UG125 (GenBank accession: PQ720541), yellow fever virus (GenBank accession: NC_002031.1), and West Nile virus (GenBank accession: NC_009942.1) generated by ViennaRNA WebServers using minimum free energy prediction. Supplementary Figure 2. CT and ISE6 supported replication of other flaviviruses. CT and ISE6 were infected with either West Nile Virus, ENTV or Powassan virus at an MOI of 0.01. The supernatant was collected 0, 3, and 6 dpi, frozen at −80°C, and titrated on BHK cells. Supplementary Figure 3. qRT-PCR RNA standard curves. ~1,000nt on NS5 containing qPCR primer binding sites was PCR-amplified with a reverse primer and a forward primer containing T7 promoter sequence. RNAs were synthesized by AmpliScribe T7 polymerase (BioSearch Technologies) following the manufacturer’s protocol. RNAs were quantified using Qubit RNA BR (Invitrogen), serially diluted to each concentration, and stored at −80°C. Supplementary Table 1. The list of primers used for the construction of infectious clone Supplementary Table 2. The list of virus abbreviations and the NCBI accession numbers [file media-1.pptx]

## Slide 1
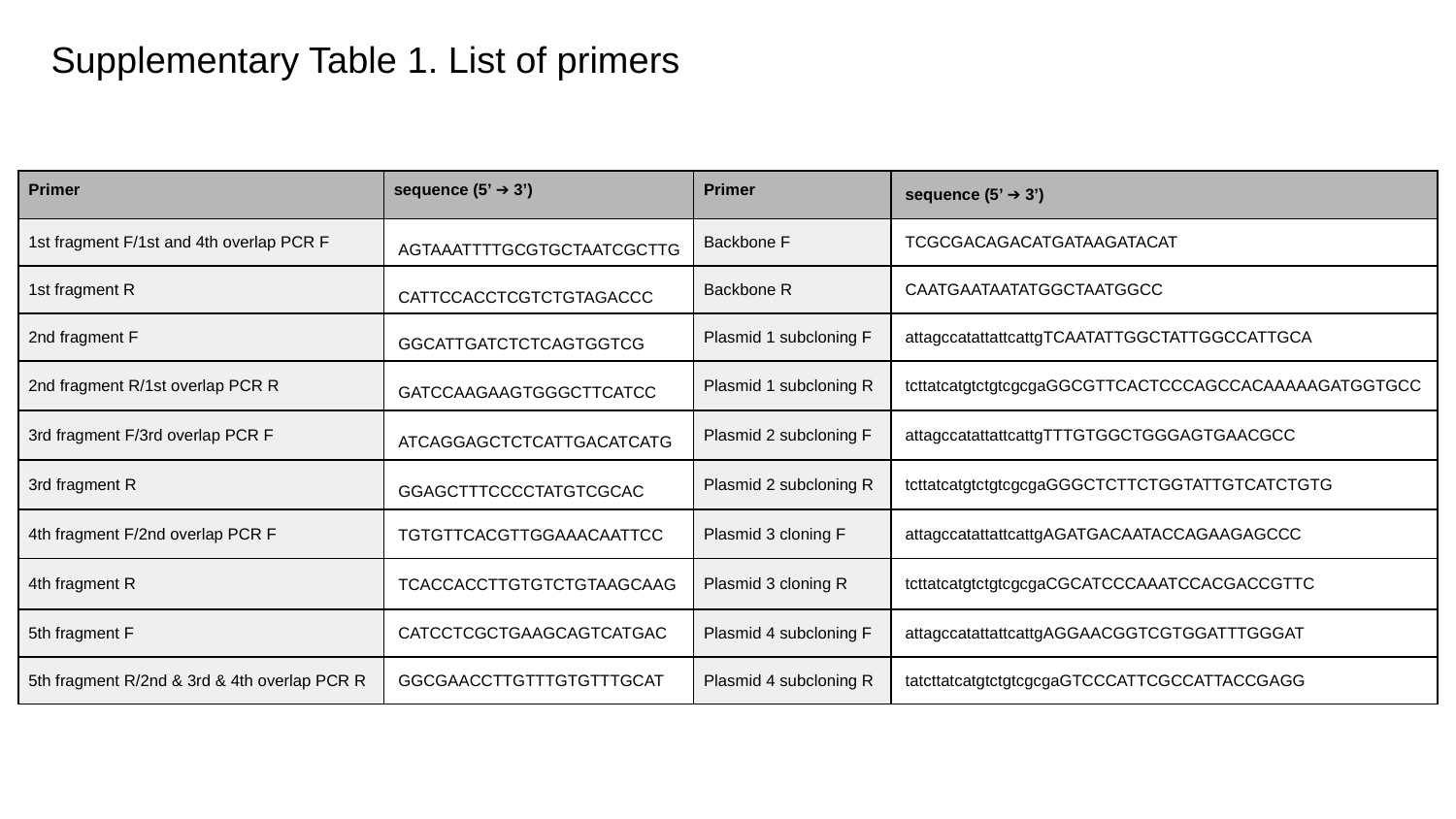

# Supplementary Table 1. List of primers
| Primer | sequence (5’ ➔ 3’) | Primer | sequence (5’ ➔ 3’) |
| --- | --- | --- | --- |
| 1st fragment F/1st and 4th overlap PCR F | AGTAAATTTTGCGTGCTAATCGCTTG | Backbone F | TCGCGACAGACATGATAAGATACAT |
| 1st fragment R | CATTCCACCTCGTCTGTAGACCC | Backbone R | CAATGAATAATATGGCTAATGGCC |
| 2nd fragment F | GGCATTGATCTCTCAGTGGTCG | Plasmid 1 subcloning F | attagccatattattcattgTCAATATTGGCTATTGGCCATTGCA |
| 2nd fragment R/1st overlap PCR R | GATCCAAGAAGTGGGCTTCATCC | Plasmid 1 subcloning R | tcttatcatgtctgtcgcgaGGCGTTCACTCCCAGCCACAAAAAGATGGTGCC |
| 3rd fragment F/3rd overlap PCR F | ATCAGGAGCTCTCATTGACATCATG | Plasmid 2 subcloning F | attagccatattattcattgTTTGTGGCTGGGAGTGAACGCC |
| 3rd fragment R | GGAGCTTTCCCCTATGTCGCAC | Plasmid 2 subcloning R | tcttatcatgtctgtcgcgaGGGCTCTTCTGGTATTGTCATCTGTG |
| 4th fragment F/2nd overlap PCR F | TGTGTTCACGTTGGAAACAATTCC | Plasmid 3 cloning F | attagccatattattcattgAGATGACAATACCAGAAGAGCCC |
| 4th fragment R | TCACCACCTTGTGTCTGTAAGCAAG | Plasmid 3 cloning R | tcttatcatgtctgtcgcgaCGCATCCCAAATCCACGACCGTTC |
| 5th fragment F | CATCCTCGCTGAAGCAGTCATGAC | Plasmid 4 subcloning F | attagccatattattcattgAGGAACGGTCGTGGATTTGGGAT |
| 5th fragment R/2nd & 3rd & 4th overlap PCR R | GGCGAACCTTGTTTGTGTTTGCAT | Plasmid 4 subcloning R | tatcttatcatgtctgtcgcgaGTCCCATTCGCCATTACCGAGG |

## Slide 2
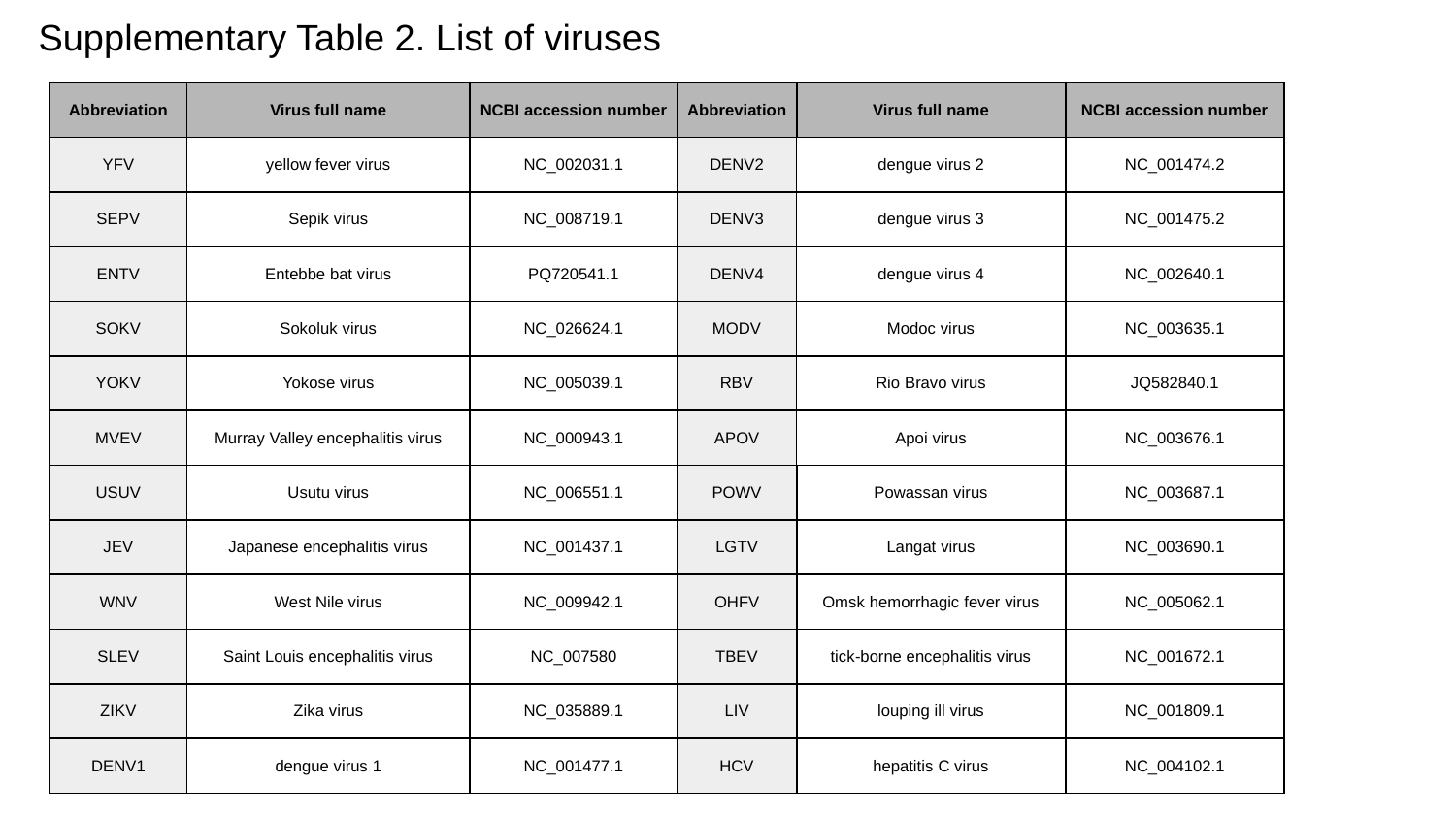

# Supplementary Table 2. List of viruses
| Abbreviation | Virus full name | NCBI accession number | Abbreviation | Virus full name | NCBI accession number |
| --- | --- | --- | --- | --- | --- |
| YFV | yellow fever virus | NC\_002031.1 | DENV2 | dengue virus 2 | NC\_001474.2 |
| SEPV | Sepik virus | NC\_008719.1 | DENV3 | dengue virus 3 | NC\_001475.2 |
| ENTV | Entebbe bat virus | PQ720541.1 | DENV4 | dengue virus 4 | NC\_002640.1 |
| SOKV | Sokoluk virus | NC\_026624.1 | MODV | Modoc virus | NC\_003635.1 |
| YOKV | Yokose virus | NC\_005039.1 | RBV | Rio Bravo virus | JQ582840.1 |
| MVEV | Murray Valley encephalitis virus | NC\_000943.1 | APOV | Apoi virus | NC\_003676.1 |
| USUV | Usutu virus | NC\_006551.1 | POWV | Powassan virus | NC\_003687.1 |
| JEV | Japanese encephalitis virus | NC\_001437.1 | LGTV | Langat virus | NC\_003690.1 |
| WNV | West Nile virus | NC\_009942.1 | OHFV | Omsk hemorrhagic fever virus | NC\_005062.1 |
| SLEV | Saint Louis encephalitis virus | NC\_007580 | TBEV | tick-borne encephalitis virus | NC\_001672.1 |
| ZIKV | Zika virus | NC\_035889.1 | LIV | louping ill virus | NC\_001809.1 |
| DENV1 | dengue virus 1 | NC\_001477.1 | HCV | hepatitis C virus | NC\_004102.1 |

## Slide 3
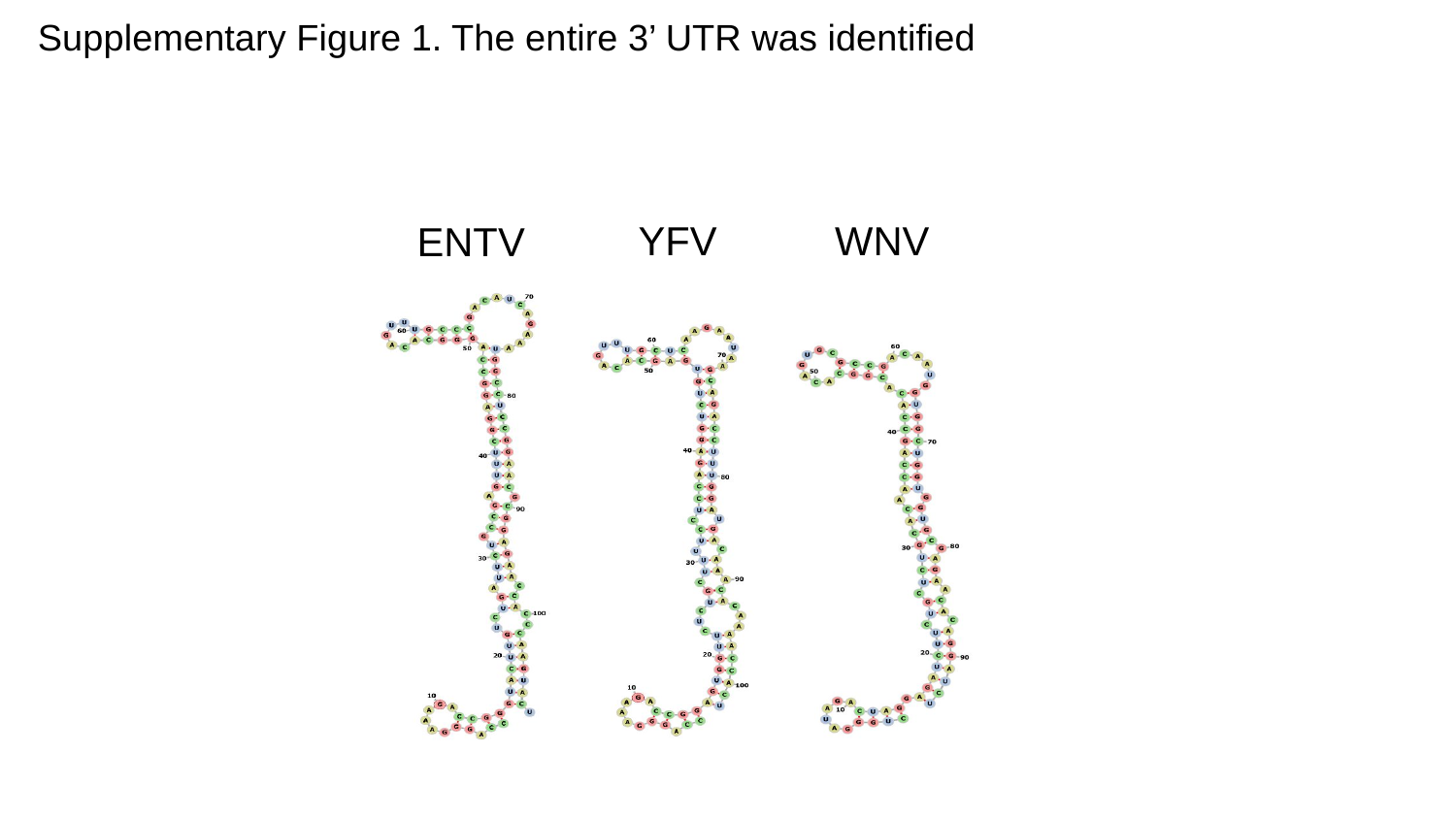

# Supplementary Figure 1. The entire 3’ UTR was identified
YFV
ENTV
WNV

## Slide 4
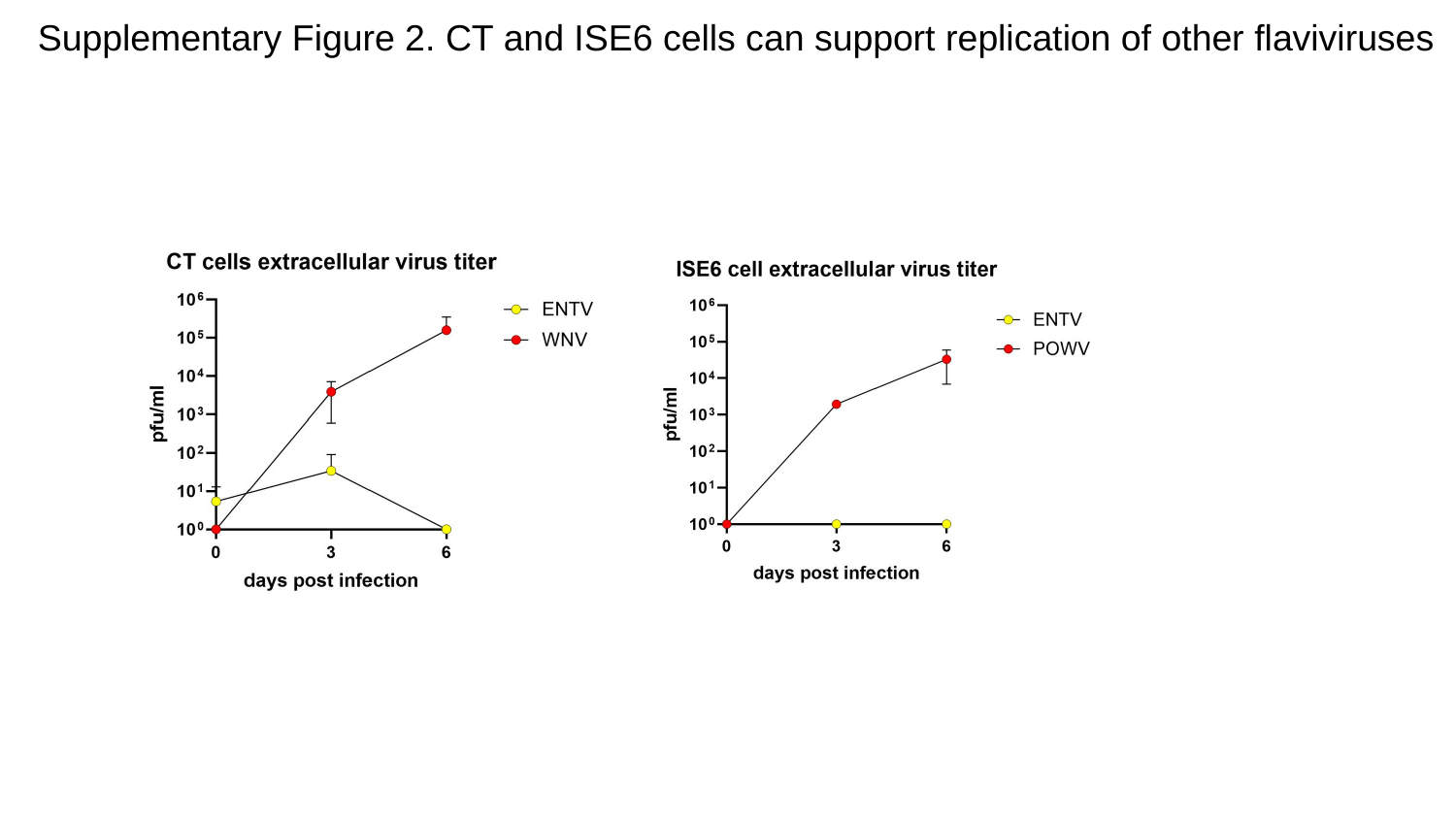

# Supplementary Figure 2. CT and ISE6 cells can support replication of other flaviviruses

## Slide 5
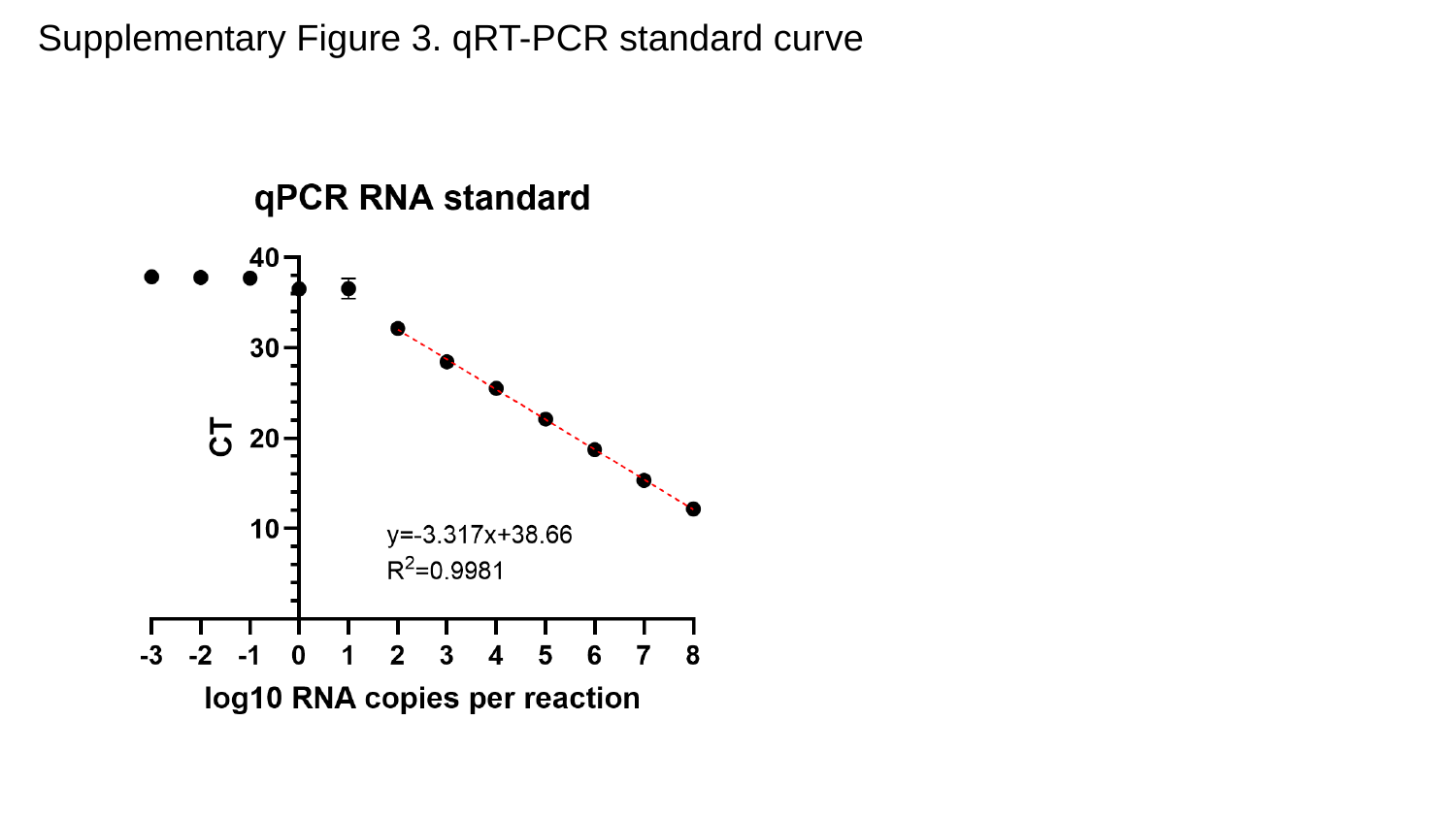

# Supplementary Figure 3. qRT-PCR standard curve
